# Disseminated Superficial Actinic Porokeratosis in a Mother and Daughter: A Case Report

**DOI:** 10.7759/cureus.80796

**Published:** 2025-03-18

**Authors:** Gayatri M Gunale, Sudhir Singh, Sabha Neazee, Sugat Jawade, Rutwik Khandeshe

**Affiliations:** 1 Dermatology, Datta Meghe Medical College, Datta Meghe Institute of Higher Education and Research (Deemed to be University), Nagpur, IND; 2 Dermatology, Jawaharlal Nehru Medical College, Datta Meghe Institute of Higher Education and Research (Deemed to be University), Wardha, IND

**Keywords:** cornoid lamella, disseminated superficial actinic porokeratosis (dsap), disseminated superficial porokeratosis, mother and daughter, porokeratosis

## Abstract

Porokeratosis is an autosomal dominant condition of epidermal keratinization. There are many clinical and morphological variants of porokeratosis, the most prevalent of which is disseminated superficial actinic porokeratosis (DSAP), which commonly affects photoexposed areas. Here, we present a 52-year-old woman and her 24-year-old daughter having similar complaints of gradually enlarging, annular hyperpigmented plaques on photoexposed areas suggesting familial transmission. Diagnosed as DSAP, treatment with oral isotretinoin and topical keratolytics showed significant improvement within one and a half months. This case report highlights familial DSAP cases in a mother and daughter, which is rare, emphasizing the importance of early diagnosis and management for better outcomes and reduced malignancy risk.

## Introduction

Porokeratosis (PK), first described by Vittorio Mibelli in 1889, is a keratinizing disorder characterized by atrophic annular lesions with a keratotic ridge [1]. It has a prevalence of 1%-4% in the general population, rising to 10%-20% in high-risk groups like organ transplant recipients. PK typically affects adults aged 40-70 years, with a female-to-male ratio of 2:1, and is more common in fair-skinned individuals, especially in areas with high ultraviolet (UV) radiation. Familial cases account for 20%-40%, suggesting a genetic component. Clinical variants include disseminated superficial PK (DSP), classical PK of Mibelli (PM), disseminated superficial actinic PK (DSAP), linear PK (LP), palmaris et plantaris PK disseminated (PPPD), PK ptychotropica, and eruptive disseminated PK [2]. DSAP is the most typical of these several clinical forms. Numerous morphological variations have been identified, including hypertrophic, gigantic, punctate, and reticulate PK [3]. DSAP is the most common variant, with lesions often appearing on photoexposed areas as brownish or reddish papules and plaques surrounded by a keratotic ridge. These lesions expand centrifugally, forming uneven or annular shapes [4]. Diagnosis is based on clinical appearance, dermoscopic findings of a characteristic ridge, and histopathology. Key histological features include a cornoid lamella, hyperkeratosis, parakeratosis, acanthosis, elongation of rete ridges, mild inflammatory infiltrates, increased apoptosis, and loss of the granular cell layer [5,6]. This case report presents a rare case of familial transmission of DSAP.

## Case presentation

**Figure 1:**
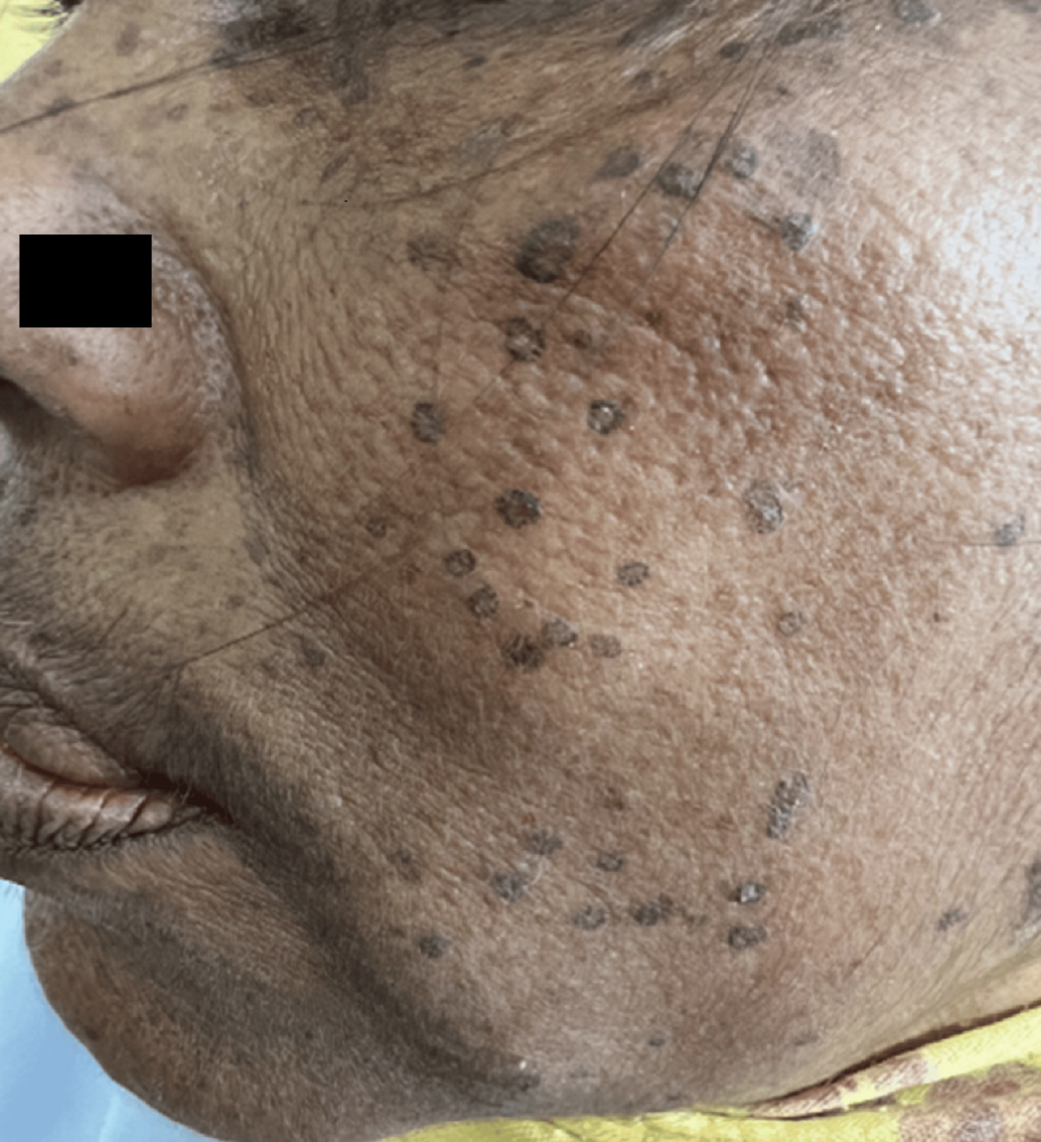
Multiple hyperpigmented annular plaques over the face

**Figure 2:**
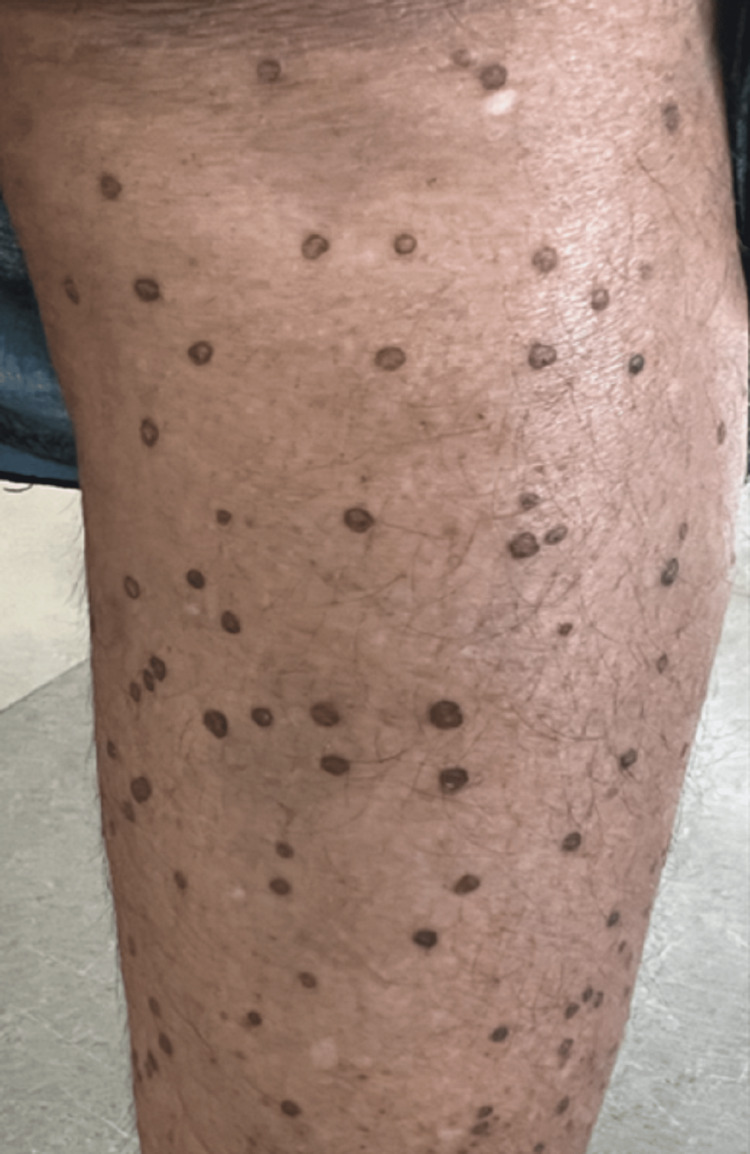
Multiple hyperpigmented annular plaques over the lower limb

A 52-year-old female patient presented to the Dermatology Outpatient Department (OPD) of a tertiary care hospital with numerous dark-colored asymptomatic raised lesions on the face, upper limbs, and lower limbs for four years. Initially, few lesions developed on the face and arms, gradually progressing to involve the lower extremities over a period of six to eight months with no signs of self-healing. Lesions increased in number and size over a period of 10-12 months. Most of the lesions were present in the photoexposed part and were symmetrical in distribution (Figures 1, 2).

**Figure 3:**
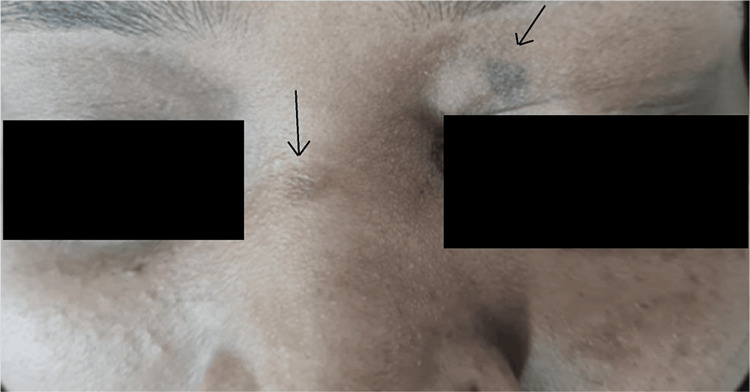
Single hyperpigmented macule over the left eyelid

**Figure 4:**
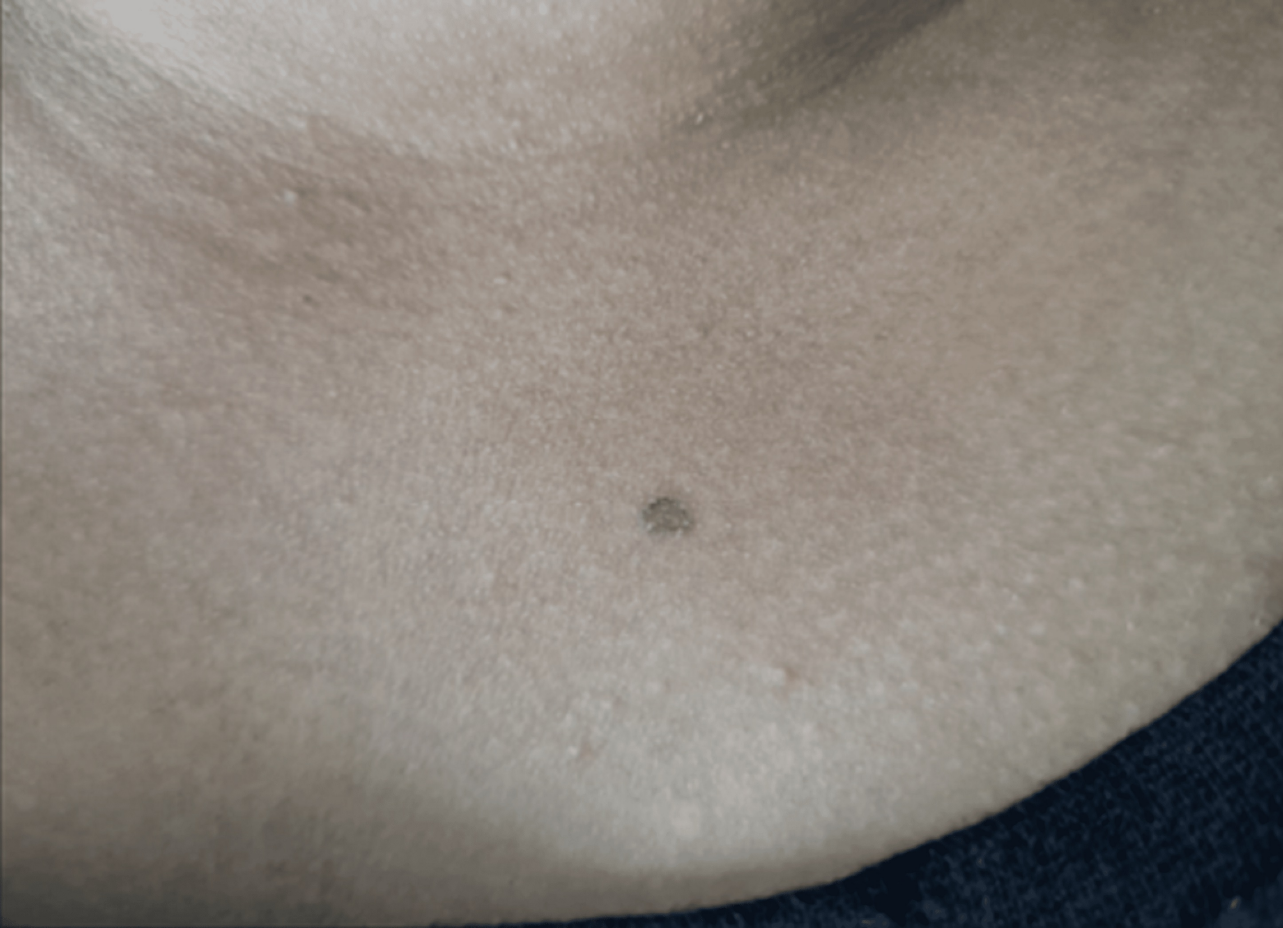
Single hyperpigmented macule over the chest

She had a history of photosensitivity. She did not have a history of any other skin lesions or recurrent infections and no history of any other comorbidities. On further inquiry, she also mentioned her daughter's history of having such lesions in the form of hyperpigmented macules over the left eyelid, bridge of the nose, and chest (Figures 3, 4). She was called in for treatment and showed partial improvement with topical retinoic acid cream. The patient came to the OPD due to a sudden increase in the size of the existing lesions for one month and an unesthetic appearance.

Cutaneous examination revealed multiple, discrete, hyperpigmented barely raised papules that combine to form different-sized annular plaques ranging from 0.3 to 2 cm in diameter with peripheral rim. The patient did not have oral lesions and nails were normal.

**Figure 5:**
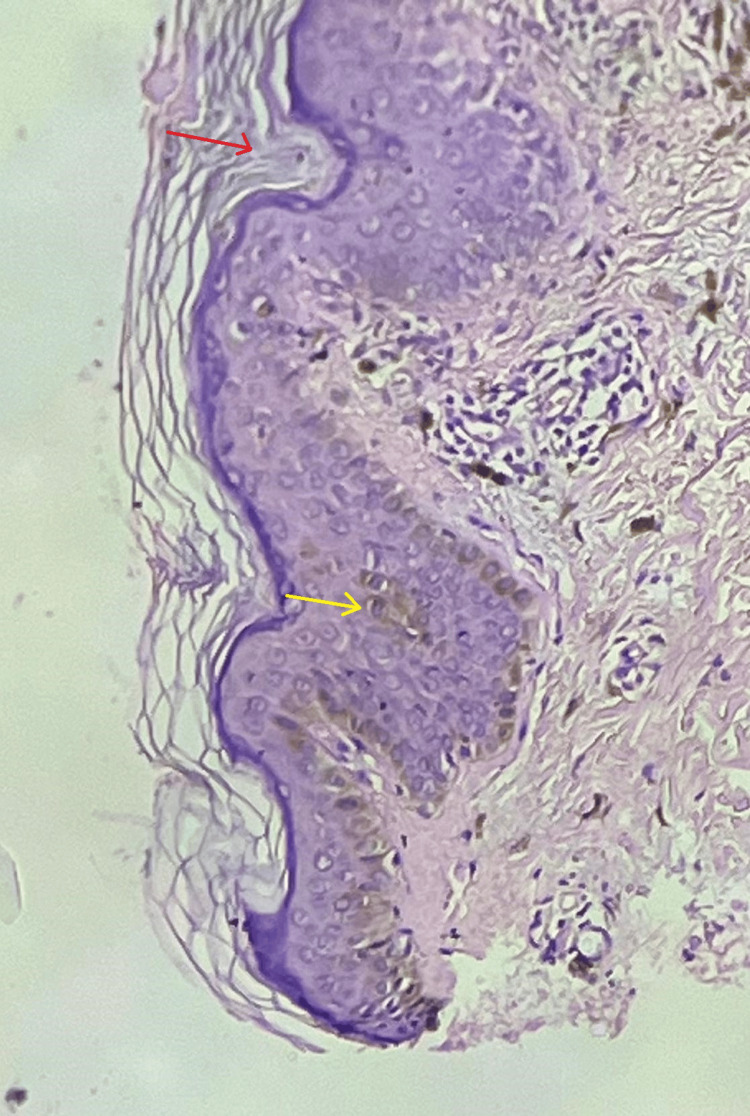
H&E stain in 10x magnification Red arrow: cornoid lamella indicating parakeratotic column; yellow arrow: acanthosis; H&E: hematoxylin and eosin

The results of the skin biopsy showed papillomatosis, acanthosis, and significant hyperkeratosis (Figure 5). There were several vertical-layered parakeratosis foci and only a few hypergranulosis foci. The bases of the parakeratotic columns had vacuolated keratinocytes and a few dyskeratotic and apoptotic cells. Melanophages and lymphohistiocytic infiltrates were observed in the upper dermis.

Clinicopathological findings led to the diagnosis of DSAP for the patient. Prior to initiating therapy, standard laboratory tests were carried out. The patient was given oral isotretinoin 20 mg at bedtime and topical keratolytics in the form of 6% salicylic acid for 20-24 weeks, and the patient was advised photoprotection.

Follow-up

The patient visited for follow-up every 15 days. There was a 40%-50% improvement in her lesions after one and a half months, and the patient was satisfied with the treatment. She was advised to continue the treatment for up to 24 weeks.

## Discussion

PK is a clonal disorder of keratinocyte growth and differentiation, rather than hyperproliferation. It manifests in various clinical forms, often linked to specific age groups. LP and PM typically emerge during childhood, while DSAP and punctate forms appear in adulthood. Palmoplantar variants generally develop during adolescence. UV exposure and immunosuppressive drugs such as cyclosporine, tacrolimus, azathioprine, mycophenolate mofetil, hydroxyurea, and fluorouracil are significant triggers for both DSAP and PM [7,8].

Genetically, DSAP is associated with mutations in SART3, SNIP1, EVER1, and EVER2, as well as chromosomal abnormalities on 16q and 3p. It often follows an autosomal dominant inheritance pattern. Contributing genetic predispositions include sensitivity to UV radiation, impaired DNA repair mechanisms, and variations in immune response genes [9].

The clinical variants of PK include distinct presentations. PK of Mibelli features hyperkeratotic, annular papules with a peripheral ridge and central atrophy [10]. LP presents as PM-like lesions distributed along Blaschko lines and has a higher risk of malignant transformation [11]. Punctate PK consists of small, hyperkeratotic macules on the palms and soles [12]. DSAP is characterized by symmetrical, threadlike, elevated rings on sun-exposed skin, typically developing after the age of 30; the non-actinic form is more commonly seen in immunosuppressed patients [2,13]. PPPD affects the palms and soles, presenting as superficial lesions with a prominent peripheral ridge [14]. Histopathological examination of PK reveals the characteristic cornoid lamella, a thin parakeratotic layer. Dermoscopic findings include central pigmentation, blue-gray specks, and a white track [15,16].

DSAP carries a 7%-11% lifetime risk of progression to squamous cell carcinoma (SCC), with an annual transformation rate of 0.5%-3.5%. Prolonged disease duration, larger lesions, cumulative UV exposure, genetic mutations, and immunosuppression are key risk factors for malignancy. Treatment options for PK include topical therapies, systemic medications, and procedural interventions. Topical treatments such as 5-fluorouracil, retinoids, tacrolimus, and diclofenac 3% gel are commonly used. Systemic retinoids, including isotretinoin and acitretin, are effective, particularly for high-risk individuals and immunocompromised patients [17,18]. Procedural options include cryotherapy, photodynamic therapy, laser therapy, and surgical excision. Topical or systemic retinoids and laser therapy have shown excellent results in managing LP, while diclofenac gel is a safe option for DSAP. For cancer prevention, oral retinoids are recommended for patients with linear or disseminated forms of the disease or those at high risk of malignancy [2,19,20].

## Conclusions

DSAP is a rare skin condition primarily seen in individuals over 50, characterized by multiple circular, hyperkeratotic plaques on sun-exposed areas. This case report highlights a unique instance of DSAP in a mother and daughter, underscoring its hereditary nature. Early diagnosis and effective treatment are essential for managing DSAP and reducing its esthetic impact and malignancy risk. The hereditary aspect of DSAP, as demonstrated in this case, suggests a need for family screening and genetic counseling in affected individuals to avoid the risk of malignancy.
